# Loading… loading… The influence of download time on information search

**DOI:** 10.1371/journal.pone.0226112

**Published:** 2019-12-06

**Authors:** Alyssa C. Smith, Brandon C. W. Ralph, Jeremy Marty-Dugas, Daniel Smilek

**Affiliations:** Department of Psychology, University of Waterloo, Waterloo, Ontario, Canada; University of Milan, ITALY

## Abstract

When browsing online, there is considerable variation in the amount of time that one has to wait for content to appear once the link to that content has been activated (i.e., clicked). In two experiments we examined how ‘download time’–a potential barrier to information access–influences search behaviour. In both experiments, participants completed a video-watching task in which they were presented with a screen containing six clickable icons, each of which represented a unique video. When participants clicked an icon, a video would begin to load and then play. The participants’ task was to gain as much information from the videos as possible for a later memory test. Critically, however, the ‘download time’ (i.e., the time between the click on the icon and the video beginning to play) of the available videos in a given search session varied. In Experiment 1, these download times were 0 (instant), 2, or 30 seconds, and in Experiment 2, they were 5, 15, or 30 seconds. In general, we found that participants terminated and avoided videos with longer download times than videos with shorter download times. Interestingly, this effect was attenuated when the experienced download times were more similar to each other (Experiment 2) than when they were more different from each other (Experiment 1).

## Introduction

People’s opinions, decisions and future plans depend on the information they consume. In turn, information consumption depends in part on what content is *available* (e.g., on the internet, in the library), the extent to which it is *accessible* [1–2], and the level to which people *attend* to the information they access [3–4]. For example, forming an opinion regarding the best earphones to purchase might depend on the availability of information about different earphones on the internet, the ability of the individual to find and access the relevant web pages, and the person’s level of attention when reading the earphone reviews he or she discovered online. Setting aside the important issues of information availability and the process of attending to selected material, here we focus on the issue of information access; and in our treatment of information access we further restrict our inquiry to the context of the World Wide Web, which is perhaps one of the fastest growing repositories of information on the planet.

Several factors can influence the likelihood that a person will access information available online. One of these factors concerns the way information is ranked or ordered by search engines and information feeds (e.g., Twitter feeds). For instance, search engines typically order their search results based on relevance to the search terms used, with the more relevant information being placed higher earlier in the search results [5–6]. Since these principles of ranking are assumed by most users, it is not surprising that people are more likely to access information that appears higher in the search results. Importantly, this bias in accessing information is not without potential consequence. Recent work by Epstein and Robertson (2015) has shown that experimentally biasing search rankings in such a way as to rank information about one political candidate above another can influence the voting decisions of undecided voters [5]. In particular, participants asked to vote for one of two candidates report a greater intention to vote for the candidate whose information was ranked higher in the search results. Interestingly, this effect occurred even though most participants were unaware that the search results were artificially manipulated, and the effect was exacerbated when the biasing information was placed above the search outcomes in a “featured results box” [7]. Highlighting the importance of their findings, Epstein and Robertson (2015, p. E4520) concluded their article with a cautionary note about how the aforementioned search biases can be deliberately exploited if search engine providers are left unchecked [5]:

“Given that search engine companies are currently unregulated, our results could be viewed as a cause for concern, suggesting that such companies could affect—and perhaps are already affecting—the outcomes of close elections worldwide. Restricting search ranking manipulations to voters who have been identified as undecided while also donating money to favored candidates would be an especially subtle, effective, and efficient way of wielding influence.”

Social media platforms have also expressed their plan to restrict (or have confessed their current practice of restricting) access to information by promoting desirable content and by downranking undesirable content. In a recent interview, Twitter co-founder Jack Dorsey commented on how information can be subtly controlled in an age when it is becoming difficult to completely remove unwanted voices from internet platforms like Twitter [8]. One of Dorsey’s “bunch of tools” for controlling information flow is to downrank certain tweets in the feed (i.e., placing them lower on the list), so that they are less likely to be noticed (these tend to be tweets that are flagged by an algorithm as reflecting aggressive behavior).

The effectiveness of downranking information as a tool for influencing information access can be understood from extant models of information foraging [9–12]. Information foraging models assume that 1) desired information is distributed unequally (on the internet, for instance) and occurs in clusters called ‘patches’; and 2) information searchers have to make decisions about whether to persevere within a particular information cluster or switch to a different cluster [13]. ‘Diet models’ of information foraging seek to explain search behaviour through the lens of profitability–that is, in terms of information gained per unit of time/energy cost. The assumption is that people seek to maximize the amount of quality information they gain (i.e., their “information diet”) while at the same time keeping their costs (e.g., time to find the information) as low as possible [11,13]. From this perspective, down-ranking decreases the profitability of select informational sources (i.e., patches) by increasing the amount of time and energy required to locate the down-ranked source. With many other potential information sources available, people ought to be more likely to ignore downranked information in favour of more readily available alternatives. In addition to modifying the ‘informational diet’, down-ranking might also impact ‘informational scent’. Information scent models describe search behaviour according to cues that might suggest higher and lower yield sources (i.e., patches) of information. Like informational diet models, informational scent models stipulate that people will follow cues with the greatest promise of return for their investment. Based on implicit assumptions and explicit statements about technologic search algorithms, downranked sources might be perceived as lower in informational quality and/or yield, and should therefore be less sought after. Thus, ranking is an effective tool for influencing information access both because it 1) influences profitability by manipulating time-costs and 2) provides cues that typically signal levels of information quality/yield.

Based on information foraging theory (and some intuition), another factor that may influence the likelihood that a person will access information online is simply the time required to access information (without down-ranking). In the realm of online human-computer interactions, an important factor might be the time it takes to download web-based information (e.g., a webpage or a video). Information foraging theory would suggest that increasing download time of a web page would increase the cost associated with the information on the web page, and this would decrease the profitability of that page. As a result, information foragers might opt to terminate a download perceived to be too costly in favor of pursuing other, possibly more profitable, prospects. Consistent with this conceptualization, the available evidence suggests that longer delays to accessing online content have been associated with reduced user satisfaction [14–15], unfavourable attitudes towards future use or revisiting of such content [16], and reduced commerce [17–18]. Importantly, increased delays to access information have been associated with increased self-reported intentions to abort access altogether [19]. Given these findings, it is perhaps not surprising that many developers and distributers seek to minimize access delays to promote access to their information or product.

The issue modulating information transfer speed (i.e., download time) as a means of controlling online information access is central to recent debates regarding Net Neutrality. One issue in the Net Neutrality debate is whether internet service providers ought to have the ability to vary internet traffic speed [20–23]. Indeed, Kramer et al. (2013, p. 796) define “Strict Net Neutrality” in the following way: “Net neutrality prohibits Internet service providers from speeding up, slowing down or blocking Internet traffic based on its source, ownership or destination.” Kramer and colleagues (p. 798) note that internet service providers may be motivated to provide “faster access lanes to its customers in return for an additional fee”, to “distort downstream competition or to limit undesired or unprofitable traffic” (such as peer-to-peer communication). Setting aside the many nuances in the Net Neutrality debate, the key point we wish to make here is that the debate highlights the fact that stakeholders might have clear motivations for modulating the speed of specific internet communications to influence the likelihood that specific services or content are accessed. We hasten to add, however, that regardless of stakeholder intentional interventions, there are many inadvertent structural factors that influence internet traffic speed (e.g., network traffic, quality of network infrastructure, quality of computer technology) and as long as these structural factors are associated with specific content (videos, or perhaps specific servers), these factors might also bias the likelihood a user will access specific types of content.

Interestingly, access delays have already been implemented in at least one smartphone application designed to discourage habitual media use. In a 60-mintues interview, Ramsey Brown, the founder of Dopamine Labs, described an application his team developed (named Space), which, at the time of the interview, could be configured to create a short delay when a user tries to access various applications on his or her smartphone [24]. The application was based on the notion that delaying access will increase the tendency of access abandonment, with the altruistic goal of breaking or preventing addictive smartphone/media use.

### The present studies

Against this backdrop, in the present paper we sought to further explore how varying the time it takes to access or ‘download’ information on the internet can influences people’s tendency to access target information, even though the information is available. Our investigation builds upon prior work examining the link between download times and internet search behaviours [25–27]. As an example of this prior work we briefly consider the studies reported by Dennis and Taylor (2006), which examined how homogenous download times in a given search session affect online search behaviour [25]. Dennis and Taylor had participants search webpages that all had either a short (0.5 seconds) download time or a long (7 seconds) download time. They found that participants who experienced homogenously long (i.e., 7 second) download times prior to viewing each webpage spent more time on the downloaded webpages than individuals who experienced homogenously shorter (i.e., 0.5 second) download times in the search session. The authors speculated that participants were scrutinizing more carefully the information on pages associated with longer than shorter download times because of the increased time costs associated with moving between the more slowly loading webpages. Dennis and Taylor (2006) also found that compared to those in the shorter download time condition, participants in the longer download time condition were less likely to switch between webpages [25], likely because participants had learned that it was more time consuming to switch pages in the long (compared to the short) download condition. These findings imply that homogenously slowing download times leads people to access fewer webpages and to spend more time on the pages they do access.

Extending the work by Dennis and Taylor (2006), we were interested in examining search behaviour as participants encountered content online, but with 1) *variable* (rather than homogenous) [25–26] access delays in a given search session and 2) the ability to switch between sources during a download. In the present studies we had participants complete a video viewing task. On each trial, participants were presented with a screen containing six clickable icons, with each icon representing a video. Participants were asked to view as many of the videos as possible within a five-minute time limit. All of the videos could not be viewed in their entirety within the time limit. When an icon was clicked, the corresponding video would be queued to play. The key feature of this paradigm was that we systematically manipulated the ‘download time’ (e.g., for 0, 2 or 30 seconds) of the videos available on a given search screen (i.e., in a search session). This meant that in contrast to prior studies using homogenous download times in a given search session, we included heterogenous download times (i.e., they varied from video to video in a given search session). We defined ‘download time’ as the time elapsed between a click on the icon and the time the video started to play. During the session, participants had complete control over which video they viewed, such that participants could start and switch between videos (by clicking another icon) at any time, with the only limitations being that just one video could play or be downloading at a time, and progress through a video would reset upon switching. This allowed us to examine the relation between download time and information access under conditions in which participants are given the opportunity to switch between information sources during a download. Participants were informed that they would be tested on the contents of all of the videos after the video viewing time was completed.

With regard to participant search behaviour, we focused on how varying information download time would influence 1) the proportion of times a download was terminated/aborted after a download has been initiated, 2) the number of times participants waited through a download and actually started viewing the target content, and 3) the tendency to finish consuming information content after viewing of the content had begun. Of course, these behaviors may be correlated with each other, but we included each of these as related measures of information consumption that might be influenced by download time. We hypothesized there would be a relation between download time and download terminations, such that during longer download times individuals would be more likely to abandon the download in favor of accessing another video. Specifically, we expect that of all videos queued to download, participants will abandon a greater proportion of videos with a long download time than videos with shorter download times. Relatedly, we also expected to find a bias whereby participants access (i.e., begin to view) information from more sources with shorter download times than those sources with longer download times. Finally, based on prior work [25] we expected that increasing download time may increase the number (and rate) of videos viewed to completion.

## Experiment one

In Experiment 1, we examined how relative differences in download times influence which information people accessed in a given information search session. For our initial investigation we chose download times with a large contrast (i.e., 0s, 2s, or 30s) to establish whether the relative differences in the download times would have any impact on the participants’ video viewing behaviour. If an effect was to be found, it would be largest (and easiest to observe) with download times with marked differences. We included a 0-second download condition to create a strong contrast with the slower download times (even though a download termination is virtually impossible during a 0-second download). We also had participants complete an end-of session strategy sheet to evaluate their awareness of the download time manipulation.

## Materials and method

This study was reviewed and received ethics clearance through a University of Waterloo Research Ethics Committee (ORE#31452), and written consent was obtained from each participant. Following the recommendations of Simmons, Nelson, and Simonsohn (2012), we report how we determined our sample size, all manipulations, all measures, and all data exclusions in this study [28].

### Participants

It was determined, a priori, that we would aim to collect data from 100 participants, as this is a reasonably large sample for within-subject comparisons with a reasonable time-cost of data collection. In total, 102 undergraduate students from the University of Waterloo participated in exchange for partial course-credit. Slight overshooting of our approximated sample size was due to participants being run in groups and overscheduling because of an anticipated occurrence of ‘no shows’.

### Materials

#### Videos

Videos presented during the video viewing task were 50- to 70-second clips from Ted Talks on a wide range of topics (Geography, Physics, Psychology etc.). A full list of the thirty videos we used can be found in the Supplementary Materials (S1 Appendix).

#### Self-reported motivation

For exploratory purposes, following the 5 trials of the video viewing task, participants were asked to rate how motivated they were to watch as many videos as possible. This was done using a scale ranging from 1 to 7 (1 = not motivated at all, 7 = extremely motivated). A copy of the motivation question can be found in Supplementary Materials (S2 Appendix).

#### End of session quiz

After responding to the motivation question, participants completed a true/false quiz on the video content. The quiz consisted of one question derived from the content of each video (30 questions total). The purpose of the quiz was 1) to prevent suspicion about the download time manipulation, and 2) to motivate participants to move through as many videos as possible during each trial. A copy of the quiz can be found in Supplementary Materials (S3 Appendix).

#### End of session strategy sheets

To measure whether participants were cognizant of our manipulation of the download time prior to viewing the videos, we asked participants (at the end of the experiment; after the quiz) to describe any strategies they used while viewing the videos. A session strategy response sheet was placed face-down beside the computer prior to the beginning of the experiment (see supporting information, S2 Appendix). At the end of the experiment, participants turned over the sheet and wrote their responses by hand.

### Procedure

#### Video viewing task

Participants were tested in groups of one to five, depending on volunteer enrollment for a given session. Participants were each seated at a computer with dividers between them so they could not observe other participants’ behaviour during the experiment. Participants were given verbal instructions for the video viewing task and then completed a 1-minute demonstration of the video viewing task with the research assistant. The instructions given for the task are provided in the supporting information (S2 Appendix).

**Fig 1:**
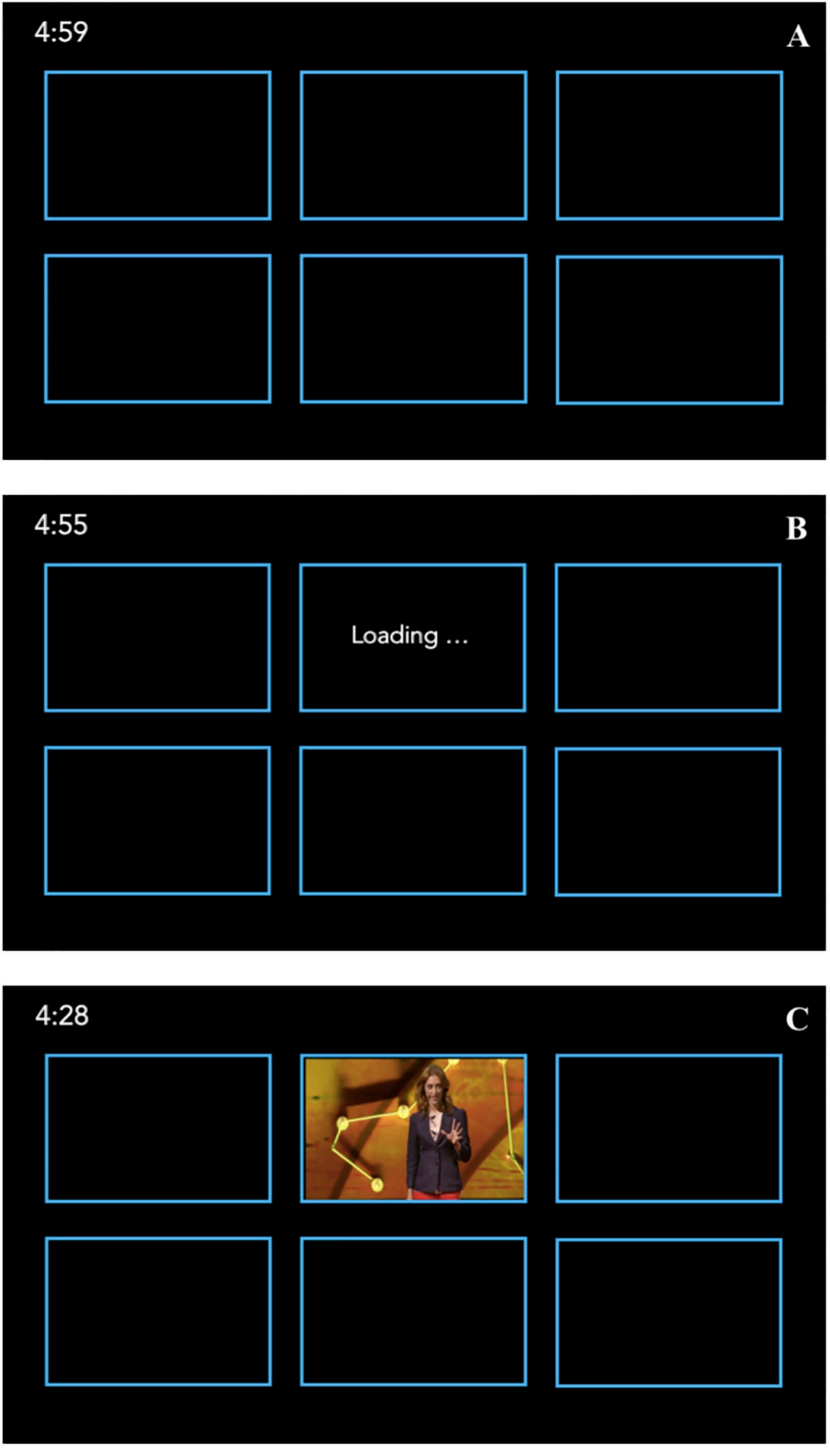
A schematic depiction of the displays that participants viewed on each trial. Included are depictions of what participants saw at the beginning of the trial (Panel A), when they clicked one of the video placeholders and the video began to load (Panel B), and when the video was playing (Panel C).

Participants completed five trials of the task. On each trial, participants were presented with six black clickable icons on the screen (Fig 1A). Each icon represented a video, and clicking an icon queued that video to play. Prior to a video starting participants could not preview the video content (unless they had already viewed a portion of that video earlier during that trial). The participants task was to view as much of the video content as possible on each trial. Participants had 5 minutes to view the videos within each trial, and there was a timer counting down from 5 minutes in the upper left corner of the screen (Fig 1).

Critically, we systematically manipulated the video download time, which we define as the time that elapsed between the video placeholder being clicked and the moment the video began to play; the download times were 0-, 2-, or 30-seconds. During the download time, participants were shown a “loading…” message to indicate the computer was processing their request (Fig 1B). No other information was provided on the length of the download time. Following the allotted download time, the video began to play (Fig 1C). To motivate participants to be strategic with regard to their video viewing choices, they were not given enough time to fully watch all six of the videos (even though they were told they would be tested on the content from all of the videos). Specifically, while participants were only given 5 minutes to watch the videos on a given trial, it would take a total of approximately 6 minutes for the videos to play sequentially. Participants could start and switch between videos at any time (even during the download) by clicking another video.

Following completion of the video viewing trials, participants were presented with the motivation question. Participants then completed a comprehension quiz on the video content.

## Results

Prior to analysis, one participant was excluded because they were given the wrong instructions, and two participants were excluded due to technical difficulties during the experiment.

### Descriptive statistics

Our three primary objectives were assessed via four key dependent variables. First, the tendency to 1) terminate/abort access during a download was measured via the *proportion of terminated downloads*, which was calculated as the number of videos that were terminated during the download time divided by the total number of videos that were queued to load (i.e., clicked). Second, the tendency to 2) wait through a download was measured via the *number of videos started*, which was calculated as the number of videos that began to play after participants had waited through the download time. Lastly, the tendency to 3) finish information content after waiting through a download was tracked via both the *number of videos finished* and the *proportion of videos finished*, which was calculated as the number of videos that were watched to the end divided by the total number of videos that started to play on a given trial. When calculating the proportion of videos finished, we excluded videos that started as the last event in a trial (as videos started as the last event in a trial would likely not have enough time left to be finished). Each of these measures was computed for each trial for the separate download time conditions (0, 2 and 30 seconds); the measures for each condition were then averaged across the 5 trials.

As can be seen in Table 1, all measures were normally distributed (skew <3, kurtosis <10; [29]) except for the proportion of terminated downloads for videos with a 0-second download time and the proportion of terminated downloads for videos with a 2-second delay, which were found to approach a non-normal distribution. Since some key variables of interest were non-normally distributed, and our data involves counts of events, we decided to analyze our key variables using non-parametric analysis techniques (Friedman test and Wilcoxon signed-rank tests). Descriptive statistics of the key variables are provided in Table 1.

**Table 1:** Descriptive statistics of variables for experiment one (N = 99).

Measure	Mean	Median	SD	Skew	Kurtosis
**Proportion of terminated downloads**^1^					
**0 seconds**	0.00	0.00	0.00	9.95	99.00
**2 seconds**	0.07	0.00	0.14	2.45	6.27
**30 seconds**	0.54	0.67	0.31	-0.7	-0.96
**Number of videos started**^2^					
**0 seconds**	1.85	2.00	0.38	-2.52	5.81
**2 seconds**	1.82	2.00	0.43	-2.43	5.41
**30 seconds**	0.56	0.20	0.75	0.93	-0.63
**Number of videos finished**^3^					
**0 seconds**	1.39	2.00	0.72	-0.74	-0.74
**2 seconds**	1.34	2.00	0.77	-0.68	-1.00
**30 seconds**	0.26	0.00	0.51	1.82	2.48
**Proportion of videos finished**^4^					
**0 seconds**	0.81	0.90	0.27	-1.56	1.44
**2 seconds**	0.79	0.90	0.28	-1.51	1.32
**30 seconds**	0.70	1.00	0.40	-0.93	-0.78

Proportions and numbers of videos averaged across 5 experimental trials

^1^Proportion of terminated downloads = the proportion of videos terminated during the download time of the total number of videos queued to load (per trial)

^2^Number of videos started = the number of videos started after the download time

^3^Number of videos finished = the number of videos finished (watched to the end) per trial

^4^Proportion of videos finished = the proportion of videos finished (watched to the end) of videos started (per trial)

Given that our dependent variables were derived from shared viewing attempts, we conducted Spearman rank-order correlations among our dependent variables for the 30-second and 2-second download time (see Table 2). We excluded the 0-second download time from our correlational analysis because terminations were nearly impossible in that condition. As can be seen in the table, the magnitude (absolute value) correlations between measures varied from .01 to .83 and the correlations varied widely between the two download time conditions. Clearly, however, none of the correlations were equal to 1, indicating that no two of our measures were identical.

**Table 2:** Correlations of dependent variables for experiment one (N = 99).

	1	2	3	4
**2-second download time**				
**1. Proportion of terminated downloads**		-.14	-.11	-.01
**2. Number videos Started**			.43*	-.20*
**3. Number videos finished**				.67***
**4. Proportion of videos finished**				
**30-second download time**				
**1. Proportion of terminated downloads**		-.83***	-.75***	-.11
**2. Number videos Started**			.76***	-.19
**3. Number videos finished**				.58***
**4. Proportion of videos finished**				

*** *p* < .001,

** *p* < .01,

* *p* < .05

### Proportion of terminated downloads

**Fig 2:**
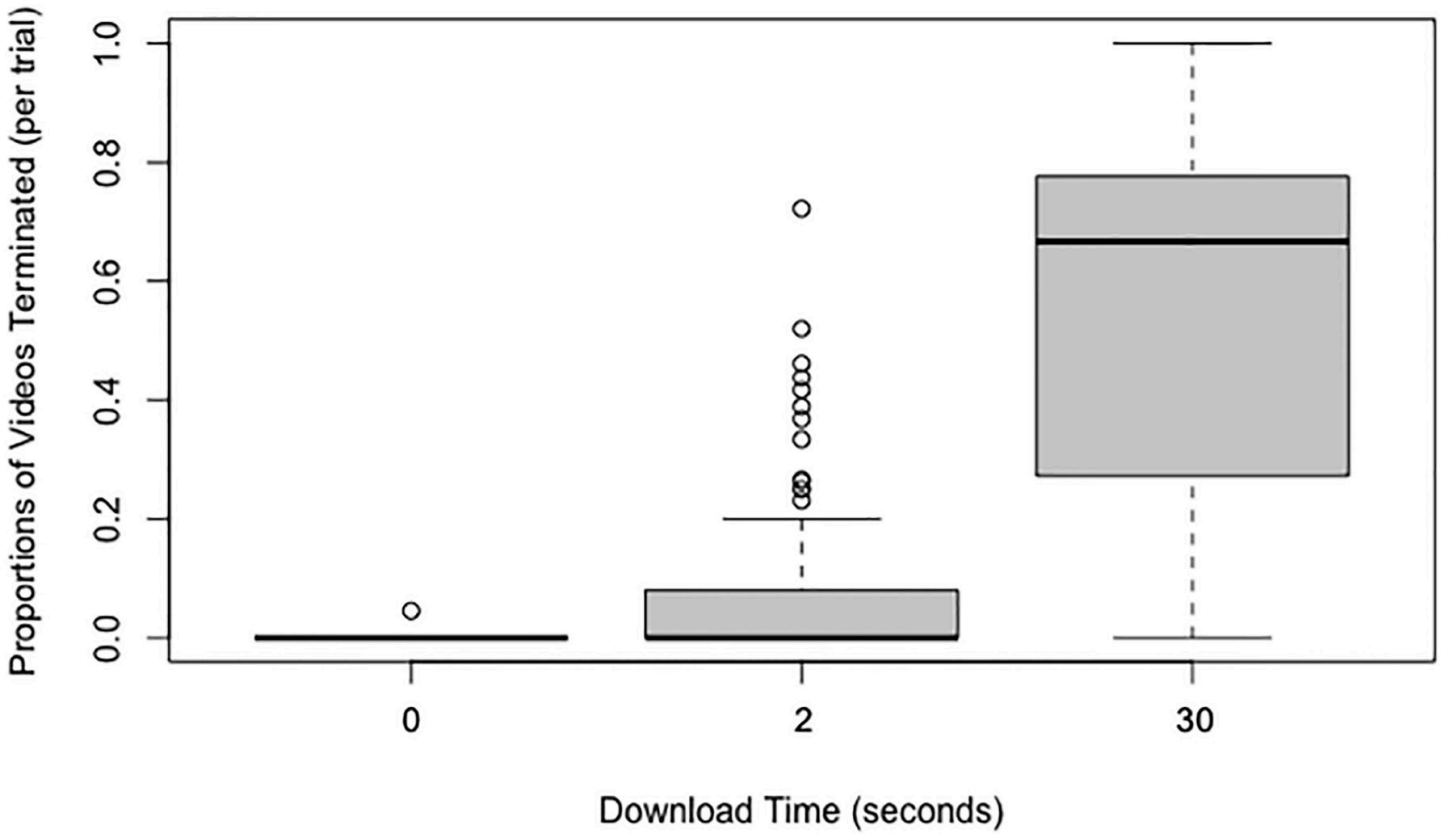
Box and whisker plots (boxplots) of the proportion of videos terminated during the download time as a function of the download time.

When analyzing the proportion of terminated downloads, we did not use an omnibus Friedman test to assess differences across all three download times because the structural constraints of the design meant that there would be zero (or near zero) terminations in the 0-second download time condition (one participant did in fact terminate a 0-second download time video. This was due to the participant clicking all the videos in rapid succession). Instead we focused on comparing the proportion of terminated downloads across the 2-second and the 30-second conditions (Fig 2). Wilcoxon signed-rank tests indicated that there were significantly more terminations during the download interval for videos with a 30-second download time than videos with a 2-second download time, *p* < .001, *r* = -0.55. For the sake of completeness, we also include the other pairwise comparisons. As might be expected, there were significant differences between videos with 30-second and 0-second download times, *p* < .001, *r* = -0.56, and also between videos with 2-second and a 0-second download times, *p* < .001, *r* = -0.34. Of course, these findings are not surprising since videos with a 0-second delay began playing immediately after being queued (i.e., clicked).

As an aside, it is worth noting that the total number of videos queued (or clicked), which served as the denominator in the calculation of the proportion of terminated downloads measure, differed across download conditions (*x*^2^(2) = 57.96, *p* < .001). Participants queued the greatest number of videos with a 30-second download time. However, when it comes to the number of first clicks on videos (to measure whether participants were disproportionately initially clicking on videos with a 30-second download time), there was no significant difference between download conditions (*x*^2^(2) = 3.01, *p* = .222). Therefore, the greater number of clicks observed on the 30-second download time videos was due to participants terminating the download, queuing another video, and then returning to the same video at a later time in the trial and queuing that video to load an additional time.

### Number of videos started to play

**Fig 3:**
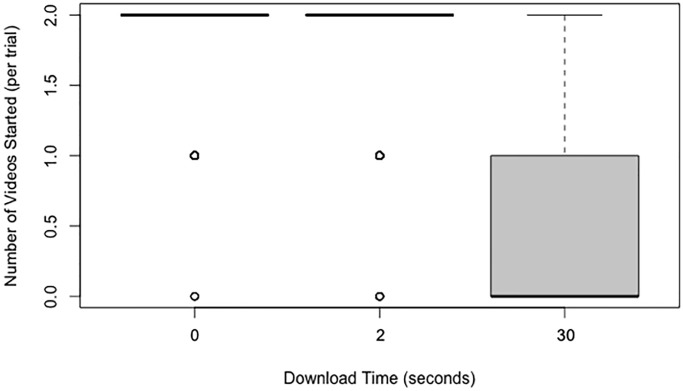
Box and whisker plots (boxplots) of the number of videos started as a function of the download time.

A Friedman test revealed that there was a statistically significant difference in the number of videos started across the three download time conditions (*x*^2^(2) = 512.96, *p* < .001; see Fig 3). Post-hoc Wilcoxon signed-rank tests indicated that there were significantly fewer videos that started to play with a 30-second download time than a 2-second download time, *p* < .001, *r* = -1.19. There were also significantly fewer videos that started to play with a 30-second download time compared to a 0-second download time, *p* < .001, *r* = -1.22. There was no significant difference between videos with a 2-second and a 0-second download time, *p* = .200, *r* = -0.09.

### Number of videos finished

**Fig 4:**
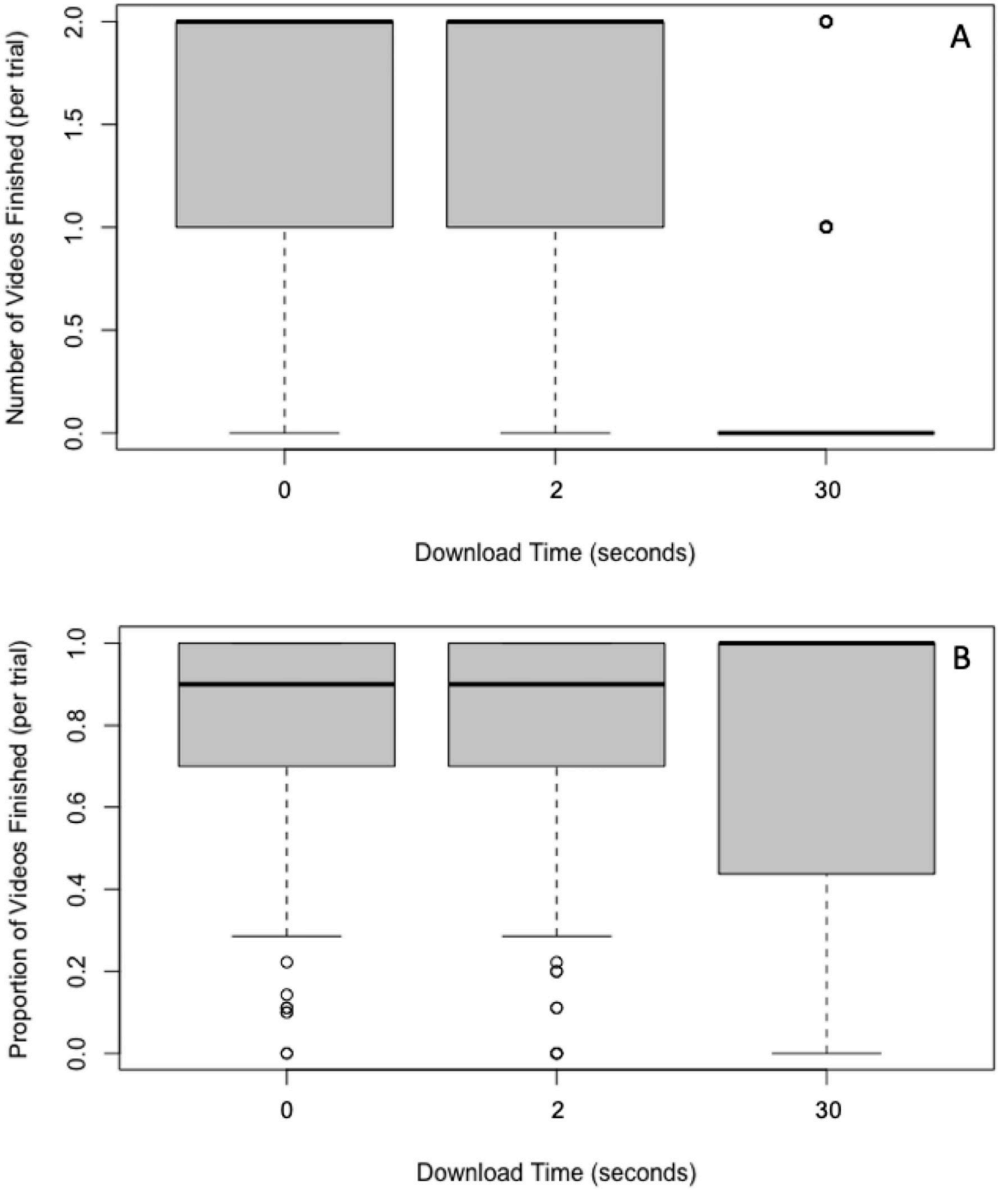
Box and whisker plots (boxplots) of the number of videos finished (A); and as a proportion of videos stared (B) as a function of the download time.

Applying the Friedman test, we found that the number of videos finished differed across the three download time conditions, (*x*^2^(2) = 100.37, *p* < .001; see Fig 4A). Post-hoc Wilcoxon signed-rank tests indicated that there were significantly fewer videos finished with a 30-second download time than a 2-second download time, *p* < .001, *r* = -0.56. There were also significantly fewer videos finished with a 30-second download time than a 0-second download time, *p* < .001, *r* = -0.57. There was no significant difference in the number of videos finished between videos with a 2-second download time and videos with a 0-second download time, *p* = .337, *r* = -0.07.

### Proportion of videos finished

Prior to analyzing the proportions of videos finished (i.e., the number of videos finished as a proportion of the number of videos started), we excluded video starts that were the last event in any given trial. Since participants only had 5 minutes to view the 6 videos in each trial, if the video start was the last event, there was not enough time left for the participant to finish that video. This excluded on average one video start per trial for each participant.

Applying the Friedman test, we found that the proportion of videos finished did not differ across the three download time conditions (*x*^2^(2) = 0.92, *p* = .631; see Fig 4B; please note that in this analysis we excluded videos that were started as the last event in any given trial, as there was not enough time for such videos to finish.).

### Motivation

We found there were some significant correlations between the video watching measures of interest and self-reported motivation. Since the self-reported motivation measure was an exploratory measure and not a main measure of interest, we included the analyses in supporting information (S4 Appendix).

### End of session strategy sheet

Forty participants (40%) noted a difference in download times between videos, and 3 of those 40 participants indicated they suspected that download time was varied intentionally. The remaining participants indicated they moved through the videos either by watching the videos that interested them or by watching a certain time-period of each video, enabling them to watch at least a small portion of each video.

### End of session quiz

Since performance on the quiz was at ceiling, we did not analyze it in relation to our video watching variables of interest. On average participants answered 86% of questions correctly, with scores ranging from 63–97%.

## Discussion

The goal of Experiment 1 was to examine how relative differences in download times influenced information access (i.e., viewing of videos) when participants had the freedom to terminate and switch between information sources, even during the download period. Our findings indicate that participants accessed more information with shorter download times than information with longer download times. This effect was consistent across several of the primary measures of interest. First, in terms of the proportion of videos terminated during download, we found participants terminated more downloads that were 30-seconds than those that were 2- or 0-seconds. Second, we examined the number of videos started (i.e. those cases in which participants waited through the download time and let the video begin to play) and found that participants started the fewest videos with a 30-second download time. Lastly, in terms of the number of videos finished, we found that compared to videos with a 2- or 0-second download time, participants finished fewer videos with a 30-second download time. However, when we computed the number of videos finished in each condition as a function of the number of videos started in each condition, the resulting proportion of videos finished did not differ across download time conditions. This suggested that the differences between conditions in the number of videos that were finished was a direct function of the differences between conditions in the number of videos that were started. Nevertheless, the data clearly show that participants consistently accessed videos with a 30-second download time less frequently than videos with a shorter download time (0-seconds, 2-seconds).

The finding that participants finished an equal proportion of videos started with a 30-second download time as those with the 2-second and 0-second download times, is somewhat surprising as it is inconsistent with prior work conducted by Dennis and Taylor (2006). Recall that Dennis and Taylor (2006) had participants complete a web search task in which a given search session included webpages that had a homogenous download times—all either short (0.5 seconds) or long (7 seconds) download times [25]. The goal of their study was to explore how the download time in a given session influenced the length of time participants spent on a webpage following its download. The authors found participants who experienced long (i.e., 7 seconds) download times prior to viewing each webpage spent more time on that webpage than individuals who experienced shorter (i.e., 0.5 seconds) download times. Based on these findings, one might expect that in our study, participants ought to finish more of the videos they started that were associated with the longer download time than the shorter download times. However, this is not what we found.

One possible explanation for this discrepancy revolves around what might be a key methodological difference across studies: Namely, Dennis and Taylor (2006) included the same download time for all webpages in a given session (i.e., a homogenous download time in a session), whereas in our study, each information gathering session (i.e., trial) included videos with a variety of download times (i.e., heterogenous download times in a session; of 0-, 2-, and 30-seconds). It is worthwhile to consider the impact of this design difference from the perspective of information foraging theory. In Dennis and Taylor’s study, the homogenous download times in a given search session might have led participants to treat the download time as a fixed component of the *switch cost* in the session; which would mean that longer download times in a session should be associated with fewer switches, and thus longer viewing times of a given webpage, in that session [25]. Because in our study the video download times in each session were heterogenous (i.e., there was no fixed switched cost) such a dynamic might not have emerged. In addition, in our study, participants were told they would be tested on the content of *all* the videos (regardless of download time), thus they may have been more motivated to continue viewing videos once they had begun since participants knew they would be tested on the content.

## Experiment two

In Experiment 2 we sought to evaluate whether the pattern of results found in Experiment 1 would change qualitatively if a different set of download times were used. The download times used in Experiment 1 included very short (0-seconds and 2-seconds) and very long (30-seconds) times, which could be perceived as being very disparate. In addition, one of the download time conditions involved no download time at all, which might have had a large impact on participant behavior. In Experiment 2 we included more similar download times (5-, 15-, and 30-second), with our short download time being non-zero. We expected to find similar patterns of results as those found in Experiment 1. Specifically, we hypothesized there would be a greater proportion of terminated downloads for videos with a 30-second download time than videos with 5-second or 15-second download times. We also hypothesized relative to the other download times, there would be fewer videos started with a 30-second download time, and fewer videos finished with a 30-second download time.

## Materials and method

### Participants

As in Experiment 1, following the recommendations of Simmons et al. (2012), we report how we determined our sample size, all manipulations, all measures, and all data exclusions in this study [28].

It was determined, a priori, that we would aim to collect data from 100 new participants (who did not participate in Experiment 1), to match the sample size in Experiment 1. In total, 94 undergraduate students from the University of Waterloo participated in exchange for partial course-credit. Slight undershooting of our approximated sample size occurred because the term ended before data from our predetermined number of participants could be collected. Instead of continuing data collection in the next term, we decided to analyze the data we had at the end of the term.

### Materials

The materials were identical to those used in Experiment 1.

### Procedure

The procedure was identical to that of Experiment 1 with the exception that the video download times were 5-, 15- and 30-seconds.

## Results

Prior to analysis, two participants were excluded due technical issues during the experiment, and one participant was excluded because they fell asleep during the experiment.

### Descriptive statistics

Descriptive statistics for our key measures of interest—proportion of terminated downloads, number of videos started, and number (and proportion) of videos finished—are presented in Table 3 As can be seen in Table 3, the proportion of terminated downloads for videos with a 5s download time was found to approach a non-normal distribution. All other variables were found to have a normal distribution (skew <3.0 and kurtosis <10.0; [29]). To allow comparison of results across studies, we decided to again analyze our key variables using non-parametric measures (Friedman test and Wilcoxon signed-rank tests).

**Table 3:** Descriptive statistics of variables for experiment two (N = 91).

Video download time	Mean	Median	SD	Skew	Kurtosis
**Proportion of terminated downloads**^1^					
**5 seconds**	0.03	0.00	0.06	2.66	8.27
**15 seconds**	0.04	0.00	0.08	2.66	7.83
**30 seconds**	0.12	0.00	0.21	2.11	3.87
**Number of videos started**^2^					
**5 seconds**	1.52	2.00	0.59	-0.78	-0.37
**15 seconds**	1.49	2.00	0.59	-0.70	-0.47
**30 seconds**	1.42	2.00	0.68	-0.74	-0.59
**Number of videos finished**^3^					
5 seconds	0.69	1.00	0.72	0.52	-0.92
15 seconds	0.71	1.00	0.69	0.45	-0.87
30 seconds	0.67	1.00	0.67	0.49	-0.74
**Proportion of videos finished**^4^					
5 seconds	0.62	0.75	0.38	-0.44	-1.39
15 seconds	0.66	0.80	0.37	-0.59	-1.22
30 seconds	0.66	0.80	0.38	-0.71	-1.08

Proportions and numbers of videos averaged across 5 experimental trials

^1^Proportion of terminated downloads = the proportion of videos terminated during the download time of the total number of videos queued to load (per trial)

^2^Number of videos started = the number of videos started after the download time

^3^Number of videos finished = the number of videos finished (watched to the end) per trial

^4^Proportion of videos finished = the proportion of videos finished (watched to the end) of videos started (per trial)

We again conducted Spearman rank-order correlations between our dependent variables for the 5-second, 15-second and 30-second download times. The correlations are presented in Table 4. Again, the correlations varied widely, but none of the correlations were equal to 1, indicating that the measures were not completely redundant.

**Table 4:** Correlations of dependent variables for experiment two (N = 91).

	1	2	3	4
**5-second download time**				
**1. Proportion of terminated downloads**		-.07	-.04	-.03
**2. Number videos Started**			-.23*	-.55***
**3. Number videos finished**				.83
**4. Proportion of videos finished**				
**15-second download time**				
**1. Proportion of terminated downloads**		.09	-.04	-.07
**2. Number videos Started**			-.20	-.56***
**3. Number videos finished**				.79***
**4. Proportion of videos finished**				
**30-second download time**				
**1. Proportion of terminated downloads**		-.40***	-.33*	.02
**2. Number videos Started**			-.08	-.53***
**3. Number videos finished**				.75***
**4. Proportion of videos finished**				

*** *p* < .001,

** *p* = .01

* *p* < .05

### Proportion of terminated downloads

**Fig 5:**
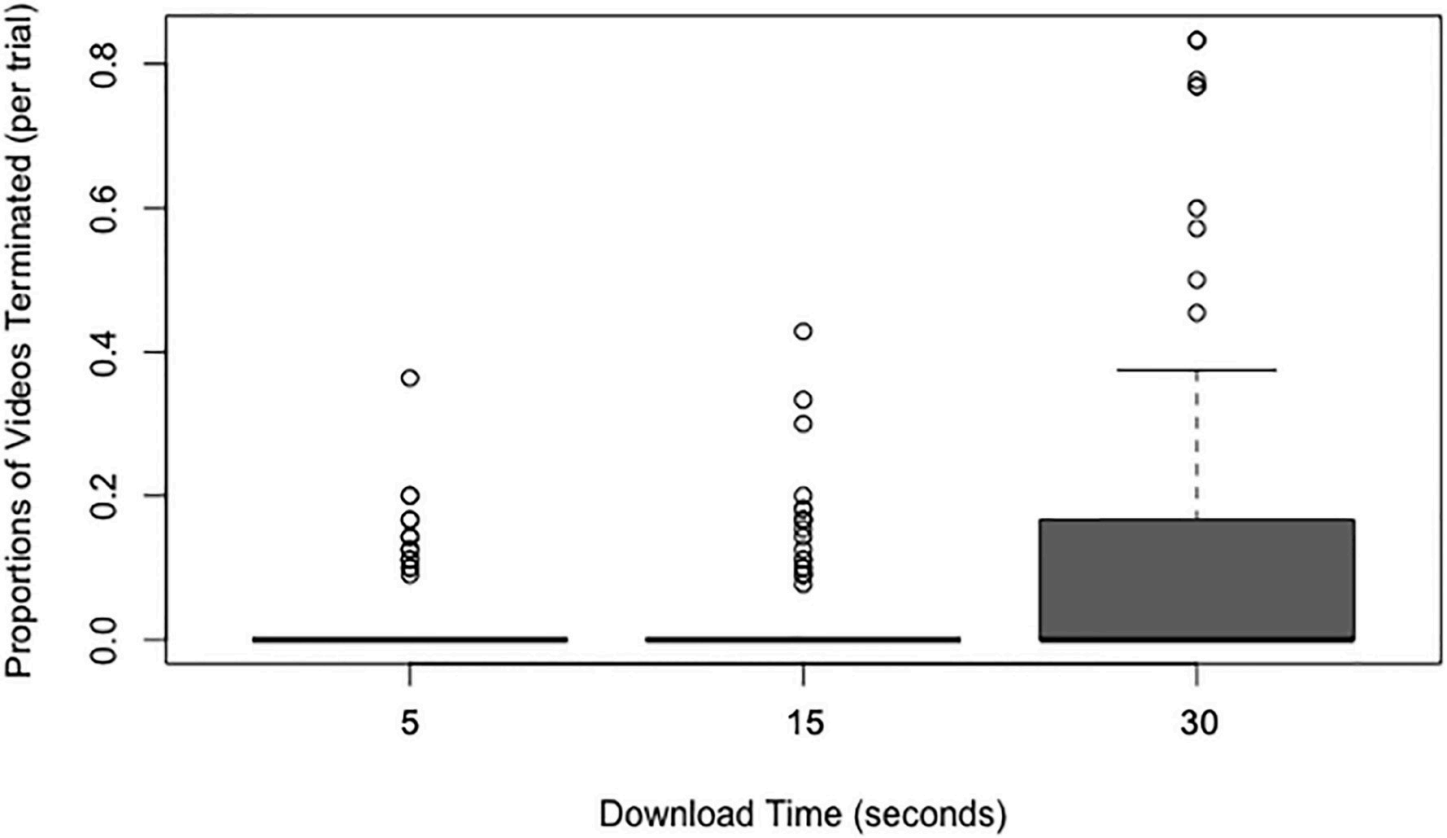
Box and whisker plots (boxplots) of the proportion of videos terminated during the download time as a function of the download time.

The results of a Friedman test revealed there was a statistically significant difference across the three download time conditions (5-, 15- & 30-seconds) in the proportion of videos terminated during the download (*x*^2^(2) = 21.93, *p* < .001; see Fig 5). Post-hoc Wilcoxon signed-rank tests indicated that there were significantly more download terminations of videos with a 30-second than a 5-second download time, *p* < .001, *r* = -0.30. There were also significantly more terminations during the download time for videos with a 30-second than a 15-second download time, *p* < .001, *r* = -0.30. There was no significant difference in the download time terminations for videos with a 15-second and a 5-second download time, *p* = .228, *r* = -0.09.

As in Experiment 1 we again analyzed the total number of videos queued and found that this differed across download conditions (*x*^2^(2) = 10.44, *p* = .005). There were again more videos with a 30-second download time that were queued to download. However, the number of first clicks on videos were roughly equivalent across download time conditions (*x*^2^(2) = 3.01, *p* = .222). Therefore, as in Experiment 1, the greater number of clicks observed on the 30-second download time videos relative to the other download time videos was due to participants terminating the download, queuing another video, then returning to the same video with the 30-second download at a later time in the trial and queuing that video to load an additional time.

Since we examined the proportion of terminated downloads for videos with a 30-second download time in both Experiment 1 and Experiment 2, we can compare the rates of terminated downloads for videos with a 30-second download time in the context of more similar (Experiment 2) and dissimilar (Experiment 1) download times. Wilcoxon signed-ranked tests indicated participants terminated significantly more videos with a 30-second download time in Experiment 1 than in Experiment 2 (*p* < .001, *r* = -0.58), with participants terminating 54% of the videos queued to download with a 30-second download time in Experiment 1 and just 12% of the videos queued to download with a 30-second download time in Experiment 2.

### Number of videos started

**Fig 6:**
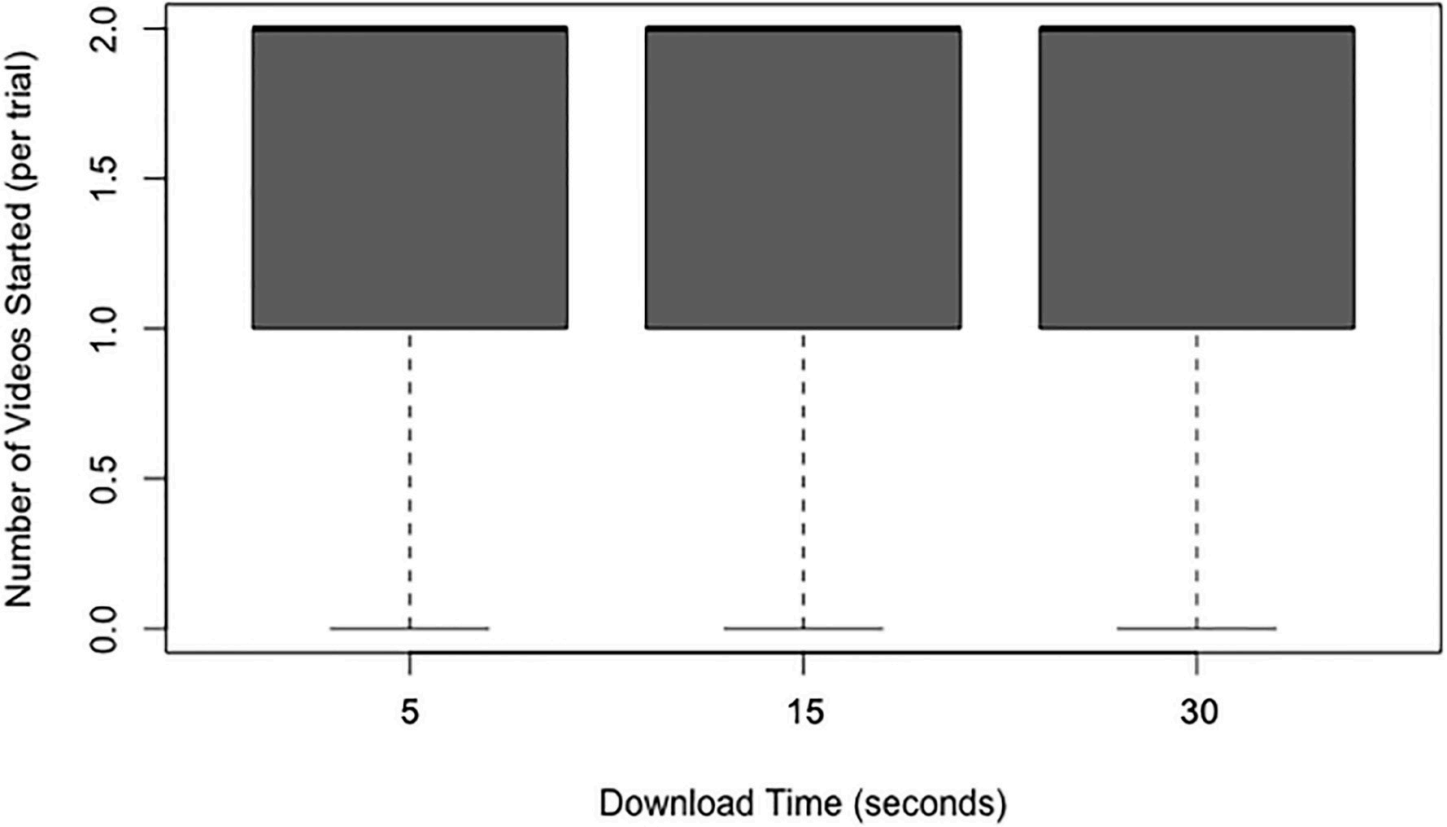
Box and whisker plots (boxplots) of the number of videos started as a function of the download time.

There was an approximately equal number of videos started across the three download times (see Fig 6). This was confirmed by a Friedman test, which showed there was no statistically significant effect of download time on the number of videos started, *x*^2^(2) = 1.80, *p* = 0.407.

### Number of videos finished

**Fig 7:**
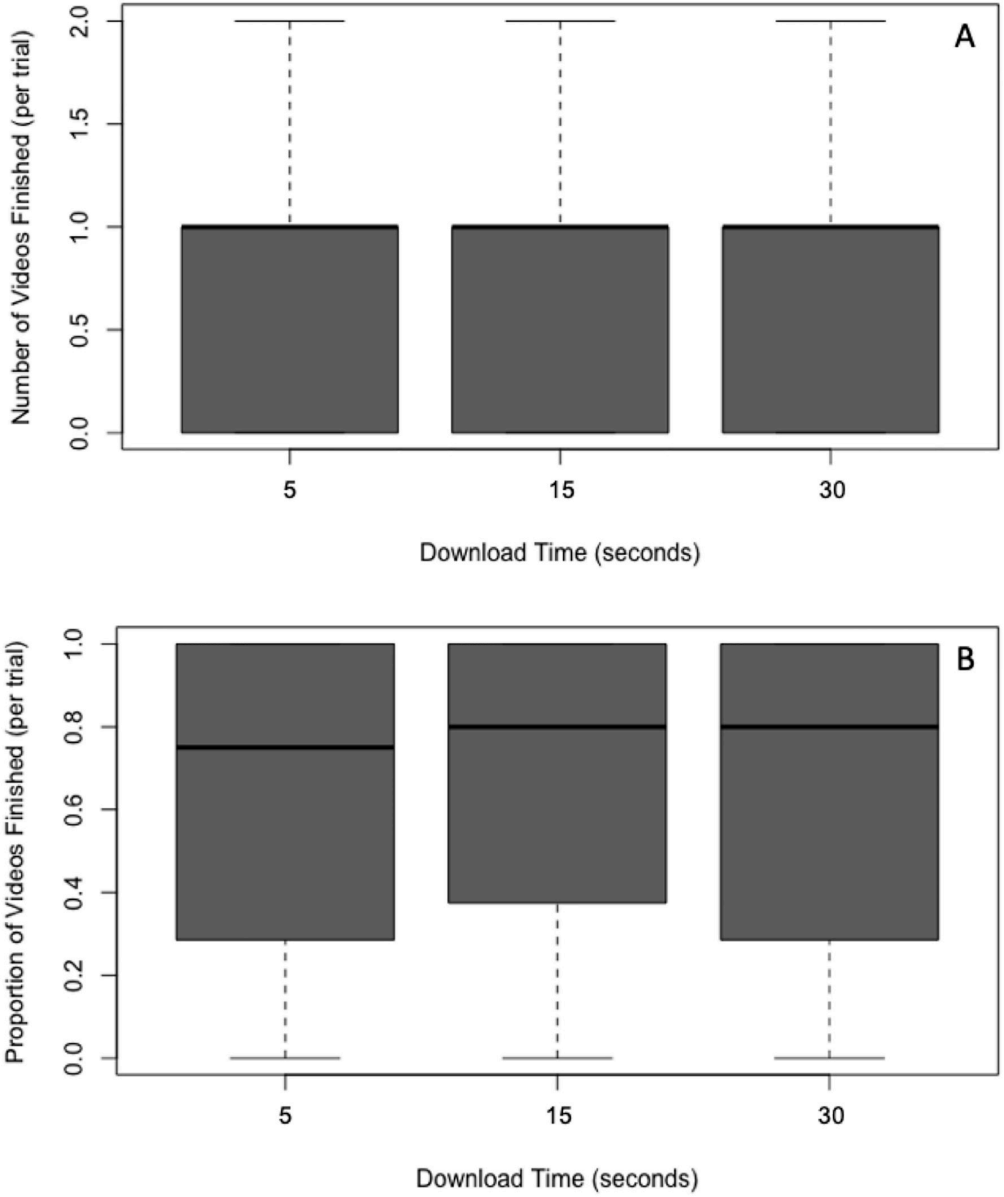
Box and whisker plots (boxplots) of the number of videos finished (A); and as a proportion of videos stared (B) as a function of the download time.

Applying the Friedman test, we found the number of videos finished did not significantly differ across the three download time conditions (5, 15, and 30 seconds), *x*^2^(2) = 1.09, *p* = 0.581 (see Fig 7).

### Proportion of videos finished

Fig 7B shows the proportion of videos finished (number of videos finished as a function of the number of videos started) in the three download conditions. Inspection of the figure reveals approximately equal proportions of videos finished across the three download times. This observation was confirmed by a Friedman test showing no statistically significant effect of download time on the proportion of videos finished, *x*^2^(2) = 2.54, *p* = 0.281.

### Motivation

We again found there were some significant correlations between our video watching measures of interest and self-reported motivation (see S4 Appendix).

### End of session strategy sheet

Four participants (0.04%) noted a difference in download times between videos, and no participants reported they suspected the differences in download time were intentional. The remaining participants indicated they moved through the videos either by watching the videos that interested them or by watching a certain time period of each video enabling them to watch at least a small portion of each video.

### End of session quiz

Quiz performance was again near ceiling and so we did not analyze it in relation to our video watching variables of interest. On average participants answered 90% of questions correctly, with scores ranging from 70–100%.

## Discussion

As in Experiment 1, the results of Experiment 2 demonstrate that people more often abandon long downloads than short downloads. However, in contrast to Experiment 1, in Experiment 2 (when using a different set of download times), we found that participants started and finished an equal proportion of videos regardless of the download time. One obvious question that emerges from this pattern of findings is: why do participants terminate more 30-second downloads than 5- or 15-second ones, but still start and finish an equal proportion of videos across all three download times? One possible explanation is that participants simply re-click (to re-queue) videos while they are downloading when they seem to take a long time to download. Such a behavior would lead to more re-clicks on videos associated with a 30-second download time than on videos associated with the shorter download times without influencing how many times the videos start to play or for how long they are ultimately watched.

It is worth noting that the results of Experiment 2 differed from Experiment 1 in another interesting way. Specifically, participants terminated significantly *fewer* 30-second downloads in Experiment 2 (about 12%) than in Experiment 1 (about 54%). One explanation for this difference is that participants experienced all of the download times included in Experiment 2 (5, 15, & 30 seconds) as being quite similar and generally ‘slow’ whereas participants experienced the downloads in Experiment 1 (0, 2, & 30 seconds) as being relatively more heterogenous, with the 30-second download time as being particularly slow relative to the other download times. This may have led participants to be more tolerant of the 30-second download time in Experiment 2 than in Experiment 1.

## General discussion

The primary goal of the two experiments reported in this paper was to evaluate whether increasing the download time of (simulated) online videos would discourage participants from accessing the content in those videos. Going beyond prior work [25, 26] we varied download times of videos *within* a given search session. In Experiment 1, we found participants terminated more downloads, and started and finished fewer videos with a 30-second download time than videos with shorter 0- or 2-second download times. These results suggest that increasing the download time of online content will, under at least some conditions, discourage engagement with the content.

The results of Experiment 2 led to a more nuanced and complex conclusion, however. Compared to Experiment 1, in Experiment 2 we included more homogeneous and generally longer download times and found that while participants continued to terminate more 30-second downloads relative to the shorter downloads (15- and 5-seconds), participants started and finished an equal number of videos regardless of the download time. Also, participants tolerated more of the 30-second downloads when the download times were slower and more homogenous (Experiment 2) than when they more heterogeneous and overall faster (Experiment 1). This suggests that as download times become more similar (and slow), participants become less likely to choose content based on download time and are not as discouraged by the slower download times in the session. Participants’ reports of the strategies they used in the experiments support this conclusion. Specifically, 40% of participants in Experiment 1 (faster, more heterogenous downloads) noted the differences in download times, while only a handful (less than 1%) of participants noted the difference in Experiment 2 (slower, less heterogenous downloads), which suggests that participants perceived the download times to be more similar in Experiment 2 than in Experiment 1. Thus, when the results of Experiments 1 and 2 are considered together, it becomes clear that whether variations in download times influence engagement with online content depends on contextual factors, with one of these factors being the specific variety of download times available in a given search session.

Our findings may provide one plausible explanation for why prior studies of ‘tolerable’ or ‘acceptable’ download times have led to widely varying estimates, ranging from 4 seconds to 41 seconds [14–16, 30–31]. Specifically, the dramatic variation in reports of tolerable wait time estimates may be partly due to variations in contextual factors across studies. That is, the variance in estimates might have emerged because 1) studies may have varied in terms of the variety of download times made available to participants and 2) different samples of participants might have had very different experiences with download times in their everyday lives (and these differences might have influenced their judgments and behaviors in an experimental session).

One way to construe the contextual effect we observed across Experiments 1 and 2 is in terms of a framing effect [32–33]. Framing effects occur when different contextual information included in the descriptions of the same underlying problem lead people to make different decisions. Framing effects are said to arise because different contextual information changes a decision maker’s reference point [34]. Applying this notion to the present studies, we could say that the relative download times in a given session provide a context against which the subjective experience of the duration of each download time is evaluated, and this evaluative context (i.e., the framing) was shifted across experiments (since they included different download times). For example, the 30-second download time, which was common across both experiments, might be experienced as being subjectively longer when compared to the 0- and 2-second download times in Experiment 1, than when compared to the 5- and 15-second download times in Experiment 2. This contextual difference could have led participants to judge the 30-second download time in Experiment 1 to be more aversive (or negative) than the 30-second download time in Experiment 2.

In light of our findings it would seem reasonable for future investigations to focus on exploring other contextual factors that might influence the impact of download times on participants’ engagement with the content associated with those download times. One example of such a contextual factor might be time pressure. In our studies, participants completed trials (i.e., search sessions) within a 5-minute time limit and it would be interesting to examine whether removing this time limit would change the relation between download time and content engagement. Another example of a potentially important contextual factor is the presence of ‘information scents’, such as an indication of the source of the content. It could very well be that people are willing to endure much longer download times to access information sources they deem more valid or those they think will provide information consistent with their worldview (reflecting a confirmation bias [35]).

Future research might also focus on exploring how barriers to online information access other than download time might influence information access. For example, it would be interesting to know how down-ranking of information affects ‘informational scent’ cues (in the language of information foraging theory). That is, as we noted in the Introduction, down-ranked information may be perceived as being less valuable (or less pertinent), thus discouraging access. Alternatively, down-ranking might simply make it less likely that the information will come into view. Regardless, given the admitted use of down-ranking by social media platforms (e.g., Twitter [36]), this form of information access manipulation ought to be examined further.

On a broader note, understanding the various factors that govern information access online will be quintessential as society navigates the various complexities of this mass communication tool we call the internet. It would be naive to dismiss the fact that control of information access can be used deliberately to manipulate public opinion, as has recently been suggested by Epstein and colleagues [5, 7, 37]. Indeed, it is well known that certain repressive governments already strictly control online access to information [38]. However, the practice of controlling the flow of, and access to, information in the public sphere has a long history in modern societies, not only in those typically labeled as being repressive and authoritarian, but also in those that are typically thought of as “free” democracies [39–42]. After propaganda was successfully used during the First World War by the British and American governments, influencers such as Edward Bernays (now often thought of as the “father of public relations”) and the Pulitzer Prize winning journalist Walter Lippmann, discussed (and even promoted) the use of information control to manage public opinion [39–42]. Democratic societies, they argued, would function more smoothly if information flowing to the population at large was selectively controlled by the ‘most capable’ individuals in society (e.g., 40). Along these lines, in his now classic work titled “Propaganda” (p. 37–38) [39], Edward Bernays wrote the following:

“The conscious and intelligent manipulation of the organized habits and opinions of the masses is an important element in democratic society. Those who manipulate this unseen mechanism of society constitute an invisible government which is the true ruling power of our country.”

“…it remains a fact that in almost every act of our daily lives, whether in the sphere of politics or business, in our social conduct or our ethical thinking, we are dominated by [a] relatively small number of persons …who understand the mental processes and social patterns of the masses. It is they who pull the wires which control the public mind, who harness old social forces and contrive new ways to bind and guide the world.”

As internet platforms become the new ‘public square’, the use of various information control tactics is likely only going to increase. Indeed, there already are cases of complete ‘de-platforming’ (erasing people from a given platform [43]), though such actions are often viewed as being too heavy handed, evoking generally distasteful thoughts of censorship. With the complete removal of information from the internet becoming more difficult—because of the rapid multiplication of internet platforms, and innovations like block-chain technology [which create multiple copies of content that are not easily tracked and deleted (see [8])]—technology companies and other influencers might turn to more subtle means of controlling what information enters the public consciousness; and it is at least conceivable that among these more subtle tools might be slowing information access or down-ranking undesirable information.

There are also, however, less deliberately calculating, though still important, factors that might influence information access on the internet. For example, continuing with our focus on download time, we note that download times can be affected by numerous factors, including 1) the type of internet connection used (e.g., dial-up, cable, fibre optic, 3G), which might differ in availability across geographical locations [44]; 2) the type of device used to access the internet [44], which might vary as a function of socio-economic status; and 3) the amount of traffic on particular webpages, which might depend on website popularity [44]. Thus, for many reasons—including those that are deliberately calculating and those that are inadvertent—it seems prudent for researchers to understand the factors that influence the likelihood of information access online.

## Supporting information

S1 Appendix (DOCX)

S2 Appendix (DOCX)

S3 Appendix (DOCX)

S4 Appendix (DOCX)
