# Supplementary material for: Loading… loading… The influence of download time on information search
Source: PLoS One. 2019 Dec 6;14(12):e0226112. doi: 10.1371/journal.pone.0226112 (PMC6897409; doi:10.1371/journal.pone.0226112)
Supplement: S1 Appendix — (DOCX) [file pone.0226112.s001.docx]

**Supporting information**

**S1 Appendix**

**Experimental Videos**

Atler, A. (2017, April). Why our screens make us less happy [Video file; 4:23-5:20]. Retrieved from https://www.ted.com/talks/adam_alter_why_our_screens_make_us_less_happy.

Achor, S. (2011, May). The happy secret to better work [Video file; 7:40-8:34]. Retrieved from https://www.ted.com/talks/shawn_achor_the_happy_secret_to_better_work.

Atala, A. (2009, October). Growing new organs [Video file; 0:59-2:06]. Retrieved from https://www.ted.com/talks/anthony_atala_growing_organs_engineering_tissue.

Autor, D. (2016, September). Will automation take away all our jobs? [Video file; 0:12-1:22]. Retrieved from <https://www.ted.com/talks/david_autor_why_are_there_still_so_many_jobs>

Bourelle, J. S. (2015, July). How culture drives behaviours [Video file; 5:32-6:40]. Retrieved from <https://www.youtube.com/watch?v=l-Yy6poJ2zs>.

Burchat, P. (February 2008). Shedding light on dark matter [Video file; 0:47-1:48]. Retrieved from https://www.ted.com/talks/patricia_burchat_leads_a_search_for_dark_energy.

Cain, S. (2012, February). The power of introverts [Video file; 4:08-5:08]. Retrieved from https://www.ted.com/talks/susan_cain_the_power_of_introverts.

Card, S. K., Pirolli, P., Van Der Wege, M., Morrison, J. B., Reeder, R. W., Schraedley, P. K., & Boshart, J. (2001, March). Information scent as a driver of Web behavior graphs: results of a protocol analysis method for Web usability. In *Proceedings of the SIGCHI conference on Human factors in computing systems* (pp. 498-505). ACM.

Chatterjee, A. (2016, November). How your brain decides what is beautiful [Video file; 11:45-12:41]. Retrieved from <https://www.ted.com/talks/anjan_chatterjee_how_your_brain_decides_what_is_beautiful>.

Cliff, H. (2015, December). Have we reached the end of physics? [Video file; 9:23-10:27]. Retrieved from <https://www.ted.com/talks/harry_cliff_have_we_reached_the_end_of_physics>.

Conley, C. (2010, February). Measuring what makes life worthwhile [Video file; 2:59-4:01]. Retrieved from <https://www.ted.com/talks/chip_conley_measuring_what_makes_life_worthwhile>.

Cuddy, A. (2012, June). Your body language may shape who you are [Video file]. Retrieved from https://www.ted.com/talks/amy_cuddy_your_body_language_shapes_who_you_are.

Ferriss, T. (2017, April). Why you should define your fears instead of your goals [Video file; 2:57-4:01]. Retrieved from https://www.ted.com/talks/tim_ferriss_why_you_should_define_your_fears_instead_of_your_goals

Foss, A. (2016, February). A prosecutor’s vision for a better justice system [Video file; 8:37-9:38]. Retrieved from https://www.ted.com/talks/adam_foss_a_prosecutor_s_vision_for_a_better_justice_system.

Genova, L. (2017, April). What you can do to prevent Alzheimer’s [Video file; 1:34-2:41]. Retrieved from https://www.ted.com/talks/lisa_genova_what_you_can_do_to_prevent_alzheimer_s.

Gilbert, E. (2009, February). Your elusive creative genius [Video file; 8:09-9:15]. Retrieved from <https://www.ted.com/talks/elizabeth_gilbert_on_genius>.

Hajek, L. (2014, March). What rives can tell us about the earth’s history [Video file; 6:49-7:57]. Retrieved from https://www.ted.com/talks/liz_hajek_what_rivers_can_tell_us_about_the_earth_s_history.

Herr, H. (2014, March). The new bionics that let us run, climb and dance [Video file; 5:33-6:41]. Retrieved from https://www.ted.com/talks/hugh_herr_the_new_bionics_that_let_us_run_climb_and_dance.

Kalanick, T. (2016, February) Uber’s plan to get more people into fewer cars [Video file; 3:18-4:24]. Retrieved from https://www.ted.com/talks/travis_kalanick_uber_s_plan_to_get_more_people_into_fewer_cars.

Kim, G. (2017, April). How cohousing can make us happier (and live longer) [Video file; 7:27-8:23]. Retrieved from https://www.ted.com/talks/grace_kim_how_cohousing_can_make_us_happier_and_live_longer.

Kundu, A. (2017, June). The boost students need to overcome obstacles [Video file; 2:36-3:43]. Retrieved from https://www.ted.com/talks/anindya_kundu_the_boost_students_need_to_overcome_obstacles.

Li, W. (2010, February) Can we eat to starve cancer? [Video file; 0:18-1:25]. Retrieved from <https://www.ted.com/talks/william_li>.

McGonigal, K. (2013, July). How to make stress your friend [Video file; 1:52-2:56]. Retrieved from https://www.ted.com/talks/kelly_mcgonigal_how_to_make_stress_your_friend.

MacGregor, N. (2011, July) 2600 years of history in one object [Video file; 0:16-1:18]. Retrieved from <https://www.ted.com/talks/neil_macgregor_2600_years_of_history_in_one_object>.

Meyer, P. (2011, July). How to spot a liar [Video file; 1:51-2:49]. Retrieved from https://www.ted.com/talks/pamela_meyer_how_to_spot_a_liar.

Ronald, P. (2015, March). The case for engineering our food [Video file; 3:53-4:58]. Retrieved from https://www.ted.com/talks/pamela_ronald_the_case_for_engineering_our_food.

Schwartz, B. (2005, July). The paradox of choice [Video file; 8:03-9:07]. Retrieved from <https://www.ted.com/talks/barry_schwartz_on_the_paradox_of_choice>.

Sinek, S. (2009, September). How great leaders inspire action [Video file; 2:23-3:18]. Retrieved from <https://www.ted.com/talks/simon_sinek_how_great_leaders_inspire_action>.

Smith, A. (2016, April). Why you should love statistics [Video file; 1:36-2:42]. Retrieved from <https://www.ted.com/talks/alan_smith_why_we_re_so_bad_at_statistics>.

Stafford, K. (2016, November). How human noise affects ocean habitats [Video file; 2:44-3:38]. Retrieved from <https://www.ted.com/talks/kate_stafford_how_human_noise_affects_ocean_habitats>.

Zomorodi, M. (2017, April). How boredom can lead to your most brilliant ideas [Video file; 2:51-4:01]. Retrieved from <https://www.ted.com/talks/manoush_zomorodi_how_boredom_can_lead_to_your_most_brilliant_ideas>.
