# Supplementary material for: Loading… loading… The influence of download time on information search
Source: PLoS One. 2019 Dec 6;14(12):e0226112. doi: 10.1371/journal.pone.0226112 (PMC6897409; doi:10.1371/journal.pone.0226112)
Supplement: S2 Appendix — (DOCX) [file pone.0226112.s002.docx]

**S2 Appendix**

**Video Viewing Task Instructions**

*[Instructions were provided to participants verbally prior to beginning the task.]*

During this experiment, you will have the opportunity to watch up to 30 videos over 5 experimental ‘blocks’. Each block will contain 6 video clips and be 5 minutes in length. Try to watch as many videos as you can. There is a clock in the upper left corner of the screen, so you can monitor the time remaining in each block. There will be a comprehension test at the end of the experiment to assess your knowledge of video content. You can watch the videos in any order and you are in control of starting, stopping, or switching between the videos at any time. After you click on each video, there may be a short delay as it loads and then it will play. However, only one video will load or play at a time. After 5 minutes has elapsed, any video you are playing and/or loading will automatically stop and you will be given a short break before the next block begins.

When the computer program ends, please fill out the sheet that is face down on your desk.

When the experiment is over, we will wait for everyone to finish so participants still working are not disrupted.

**Motivation Question**

*[Participants indicated their motivation on the 1-7 with a key press.]*

How motivated were you to watch as many videos as you could?

1. Not motivated at all
2. Extremely motivated

**Strategy Sheet**

*[Participants provided their answers by hand.]*

Please describe any strategies you used when viewing videos:

**______________________________________________________________________________________________________________________________________________________________________________________________________________________________________________________________________________________________________________________________**
