# Supplementary material for: Loading… loading… The influence of download time on information search
Source: PLoS One. 2019 Dec 6;14(12):e0226112. doi: 10.1371/journal.pone.0226112 (PMC6897409; doi:10.1371/journal.pone.0226112)
Supplement: S3 Appendix — (DOCX) [file pone.0226112.s003.docx]

**S3 Appendix**

**End of Session Quiz**

*[Questions were presented one at a time and appeared in a random order for each participant.]*

The comprehension test will consist questions based on video content you just had the opportunity to view. All 30 questions are TRUE / FALSE.

1. Since the introduction of the automated teller machine (ATM) the number of bank tellers in the US has increased. [Autor]
2. Research has shown boards composed of different cultures perform worse than those composed of only one culture. [Bourrelle]
3. Researchers have demonstrated individuals with facial abnormalities are thought to be less kind and less hard working. [Chatterjee]
4. We can test the idea of the multiverse, so we are reasonably certain it exists. [Cliff]
5. Maslow wanted his hierarchy of needs to be applied to the collective as well as the individual. [Conley]
6. There was an argument made by one of the speakers that it was a mistake for individuals to begin thinking they *are* a genius instead of *having* a genius. [Gilbert]
7. Capillaries (tiny blood vessels) cannot adapt to the environment (liver, lungs, muscle etc.) they’re growing in. [Li]
8. The main object of discussion in one of the videos was the shape of a rugby ball and made approximately 2500 years ago. [MacGregor]
9. When individuals are presented with more choices, they find it easier to make a choice. [Schwartz]
10. “Inspired” corporations think from the inside out (why, how, what) versus from the outside in (what, how, why). [Sinek]
11. Individuals spend a greater amount of time on the apps that make them happy than the ones that make them unhappy. [Atler]
12. The synapse is where Alzheimer’s happens. [Genova]
13. Individuals who are blind display a different physical expression of victory than those with sight. [Cuddy]
14. When looking at the components of the universe, ordinary matter makes up the smallest proportion of the universe. [Burchat]
15. When studying the Earth’s history, there is nothing in our current environment that allows us to look back on the landscapes of the past. [Hajek]
16. When examining rates of low numeracy around the world, the Netherlands and Korea have the lowest percentages of their populations with low numeracy. [Smith]
17. In ocean habitats, marine mammals see using light under water. [Stafford]
18. When you share meals with your neighbours, you start to plan more activities together and share more things. [Kim]
19. In the US, millions of hours a year are wasted sitting in traffic. [Kalanick]
20. Stoicism involves training yourself to separate what you can control from what you cannot control. [Ferriss]
21. Introversion and being shy are the same thing. [Cain]
22. Researchers have shown that your external circumstances predict only a small amount of your long-term happiness. [Achor]
23. Most rice varieties will die if submerged in water for more than 1 day. [Ronald]
24. To overcome their circumstances, the examples in the video had both grit and agency. [Kundu]
25. Lying is a solo act. [Meyer]
26. Studies have shown the belief that stress was bad for you leads to an increased risk of death. [McGonigal]
27. Studies have shown the activation of the brain’s default mode network is not related to boredom. [Zomorodi]
28. One patient dies from a disease that could be treated using tissue replacement every 30 seconds. [Atala]
29. There was no mathematical modeling used in the design and attachment of the speaker’s bionic limbs. [Herr]
30. Prosecutors cannot be told how to prosecute their cases. [Foss]
