# Supplementary material for: Loading… loading… The influence of download time on information search
Source: PLoS One. 2019 Dec 6;14(12):e0226112. doi: 10.1371/journal.pone.0226112 (PMC6897409; doi:10.1371/journal.pone.0226112)
Supplement: S4 Appendix — (DOCX) [file pone.0226112.s004.docx]

**S4 Appendix**

**Experiment One**

We were also interested in the correlation between our measures of interest and participants’ self-reports of motivation at the end of the experiment. Participants were asked to rate how motivated they were to watch as many videos as possible. This was done using a scale ranging from 1 to 7 (1 = not motivated at all, 7 = extremely motivated). Since some of our measures were had non-normal distributions, we conducted a Spearman’s Rank Order Correlation for each measure of interest and participants’ self-reports of motivation.

Overall, participants rated being reasonably motivated to watch as many videos as possible with a mean motivation of 5.30.

***Proportion of terminated downloads.*** As presented in S1 Table, there were no significant relations between participants’ self-reports of motivation and the proportion of terminated downloads for any download time.

***Number of videos started.*** As presented in S1 Table, there was a significant relation between self-reports of motivation and the number of videos started with a 0-second download time, *r*(97) = .23, *p* = .024, and the number of videos started a 2-second download time, *r*(97) = .26, *p* = .011. There was not a significant relation between self-reports of motivation and the number of videos that started with a 30-second download time, *r*(97) = .-10, *p* = .305.

***Number of videos finished.*** As presented in S1 Table, there was not a significant relation between self-reports of motivation and the number of videos finished with a 0-second download time, *r*(97) = .07, *p* = .513, and the number of videos finished with a 2-second download time, *r*(97) = .10, *p* = .322. There was a significant relation between self-reports of motivation and the number of videos finished with a 30-second download time, *r*(97) = -.25, *p* = .012. Those who are more motivated, finish significantly fewer videos with a 30-second download time.

***Proportion of videos finished.*** As presented in S1 Table, there were no significant relations between self-reported motivation and the proportion of videos finished. However, the relation between the motivation and the proportion of videos finished with a 30-second download time approached significance, *r*(97) = -.28, *p* = .054.

**S1 Table. Spearman rank order correlation between key measures and motivation by download time.**

| **Download Time** | **Proportion of terminated downloads** | **Number of videos started** | **Number of videos finished** | **Proportion of videos finished** |
| --- | --- | --- | --- | --- |
| **0 seconds** | -.17 | .23* | .07 | -.02 |
| **2 seconds** | -.10 | .26** | .10 | -.01 |
| **30 seconds** | .06 | -.10 | -.25* | -.28 |

** *p* < .01, * *p* < .05

**Experiment Two**

Participants in Experiment Two were presented the same motivation question as in Experiment One. Since again some of our measures were had non-normal distributions, we conducted a Spearman’s Rank Order Correlation for each measure of interest and participants’ self-reports of motivation.

Overall, participants in Experiment two also rated being reasonably motivated to watch as many videos as possible with a mean motivation of 5.23.

***Proportion of terminated downloads.*** As presented in S2 Table, there were no significant relations between participants’ self-reports of motivation and the proportion of terminated downloads for any download time.

***Number of videos started.*** There was not significant relation between self-reports of motivation and the number of videos started with a 5-second download time, *r*(89) = .19, *p* = .065, nor the number of videos started a 30-second download time, *r*(89) = .-.09, *p* = .371. There was however, a significant relation between self-reports of motivation and the number of videos that started with a 15-second download time, *r*(89) = .29, *p* = .004. More motivated participants finished more videos with a 15-second download time (S2 Table).

***Number of videos finished.*** There was not a significant relation between self-reports of motivation and the number of videos finished with a 5-second download time, *r*(89) = -.07, *p* = .527, nor the number of videos finished with a 15-second download time, *r*(89) = -.07, *p* = .489. There was a significant relation between self-reports of motivation and the number of videos finished with a 30-second download time, *r*(89) = -.29, *p* = .006. Such that, those who are more motivated, finish significantly fewer videos with a 30-second download time (S4 Table 2).

***Proportion of videos finished.*** As presented in S2 Table, there were no significant relations between self-reported motivation and the proportion of videos finished.

**S2 Table. Spearman rank order correlation between key measures and motivation by download time.**

| **Download Time** | **Proportion of terminated downloads** | **Number of videos started** | **Number of videos finished** | **Proportion of videos finished** |
| --- | --- | --- | --- | --- |
| **5 seconds** | **-.02** | **.19** | **-.07** | **-.06** |
| **15 seconds** | **.17** | **.29**** | **-.07** | **-.15** |
| **30 seconds** | **.19** | **-.09** | **-.29**** | **-.14** |

** *p* < .01, * *p* < .05
